# The association between violence victimization and subsequent unplanned pregnancy among adolescent girls in Uganda: Do primary schools make a difference?

**DOI:** 10.1371/journal.pgph.0001141

**Published:** 2023-07-31

**Authors:** Katherine G. Merrill, Louise Knight, Janet Nakuti, Angel Mirembe, Elizabeth Allen, Amiya Bhatia, Jenny Parkes, Dipak Naker, Karen M. Devries

**Affiliations:** 1 Center for Dissemination and Implementation Science, Department of Medicine, University of Illinois Chicago, Chicago, Illinois, United States of America; 2 London School of Hygiene and Tropical Medicine, London, United Kingdom; 3 Raising Voices, Kampala, Uganda; 4 University College London-Institute of Education, London, United Kingdom; University of Murcia, SPAIN

## Abstract

Violence victimization is a risk factor for adolescent pregnancy in high-income, low violence prevalence countries, but longitudinal data are lacking from settings where violence and adolescent pregnancy are common, including sub-Saharan Africa. We also know little about contextual factors which modify this association. We analyzed data from the Contexts of Violence in Adolescence Cohort (CoVAC) study in Luwero District, Uganda. Primary students in 42 schools completed surveys in 2014 (Wave 1) and 2018 (Wave 2). Our outcome was unplanned pregnancy. Our exposure was violence victimization, including any violence, type of violence (physical, emotional, sexual), perpetrator group (teacher, peer, family member), and polyvictimization. We fit mixed-effects logistic regression models and examined school factors (e.g., connectedness, absenteeism) as effect modifiers, using data from students (n = 3,431) and staff (n = 591) at the 42 schools. 1,449 girls were included in analyses (78% follow-up). At Wave 1, 88% (n = 1,281/1,449) reported any violence (mean age = 12.73, SD = 1.44 years). At Wave 2, 13.9% (n = 201/1,449) reported an unplanned pregnancy. In adjusted models, compared to no violence, significant associations (p<0.05) were observed for any violence (OR = 1.99, 95%CI = 1.03–3.85), physical violence (OR = 1.96, 95%CI = 1.02–3.79), teacher violence (OR = 1.96, 95%CI = 1.01–3.79), peer violence (OR = 2.00, 95%CI = 1.00–4.03), family violence (OR = 2.23, 95%CI = 1.07–4.65), violence from one perpetrator group (OR = 2.04, 95%CI = 1.01–4.15), and violence from three perpetrator groups (OR = 2.21, 95%CI = 0.99–4.95). Sexual and emotional violence were associated in crude but not adjusted analyses. School and peer connectedness modified the association (p<0.05); girls who experienced violence had higher odds of unplanned pregnancy in schools with lower versus higher connectedness. Violence victimization in early adolescence is strongly associated with subsequent unplanned pregnancy among adolescent girls in Uganda but attending schools with more school or peer connectedness attenuated this link. Interventions should seek to reduce violence against girls to prevent unplanned pregnancy. Interventions promoting positive connections to school may be especially important for violence victims.

## Introduction

Rates of adolescent pregnancy have been slow to decrease or have increased in many countries in the past thirty years [1]. Rates have remained particularly high in low- and middle-income countries (LMIC), where about 21 million girls (aged 15–19 years) become pregnant and 12 million give birth each year; roughly half of these pregnancies are unwanted [2]. Of all the world regions, sub-Saharan Africa has the highest rates of adolescent pregnancy, at 100 births per 1,000 adolescent girls [3]. In Uganda, a quarter of girls aged 15–19 years are either mothers or pregnant [4].

Early pregnancy has a host of detrimental health and social effects for adolescent girls. Girls who become pregnant in adolescence are more likely to use substances [5], acquire HIV and other sexually transmitted infections [6], and experience adverse pregnancy outcomes like unsafe abortion [7]. Pregnancy during adolescence has also been associated with negative mental health outcomes (e.g., depression, posttraumatic stress disorder) during and after the postpartum period [8]. Studies show that perinatal adolescents have higher rates of depressive symptoms than perinatal adults [9]. Moreover, complications during pregnancy and childbirth is a leading cause of death among girls aged 15–19 globally [10].

Violence victimization—referring to direct exposure to physical, sexual, emotional, or other forms of violence, distinguished from witnessing or perpetrating violence—is a recognized risk factor for pregnancy among adolescent girls and young women, based on meta-analyses from 2009 [11] and 2014 [12]. Findings on the association between violence victimization and adolescent pregnancy have been supported by more recent longitudinal [13–17] and cross-sectional [18, 19] studies. However, gaps in the literature remain. There is a need for greater research on the types of violence (e.g., physical, emotional, sexual) and polyvictimization as they relate to adolescent pregnancy [12, 20]. We also know little about the perpetrators or contexts in which violence leads to adolescent pregnancy. Furthermore, there is a paucity of longitudinal studies on the topic, especially from LMIC; of the five prospective longitudinal studies included in the 2014 meta-analysis, all were restricted to high-income countries [12], and only one longitudinal study conducted since then was identified in the literature from a LMIC (South Africa) [14].

Much less evidence exists about how practitioners and policymakers can effectively interrupt the association between violence victimization and adolescent pregnancy. This is especially true in thinking about modifiable aspects of the contexts in which adolescents spend time, such as the school and home. The school context is recognized as important for the social, emotional, and physical development and well-being of children and adolescents. Studies have found relationships between violence in schools and school factors, such as negative perceptions of school climate [21] and low school connectedness [22], high proportions of students eligible for free- or reduced-price meals [23], and low academic performance [24]. Some studies have found that teacher characteristics, such as teacher anxiety and depressive symptoms [25], are linked with the occurrence of school violence. School factors are also recognized to be associated with adolescent pregnancy. High school expectations, achievement, and engagement are protective factors for pregnancy [26, 27], while girls who become pregnant are more likely to drop out of school [28]. However, studies have yet to consider whether school factors can interrupt the association between violence victimization and adolescent pregnancy.

To fill these gaps in the literature, we examined longitudinal associations between multiple forms of violence victimization and unplanned pregnancy in a cohort of adolescent girls in Uganda. We sought to expand on existing studies by considering the type of victimization (i.e., physical, sexual, or emotional), the perpetrator group (i.e., teacher, peer, or family member), and polyvictimization (i.e., violence from multiple perpetrator groups) as they relate to adolescent pregnancy. We also assessed whether any primary school factors (e.g., school connectedness, school climate, etc.) may modify the association between experiences of violence and unplanned pregnancy, with the view toward developing strategies to interrupt the pathway if identified.

## Methods

### Study design and sample

Data were analyzed from the Context of Violence in Adolescence Cohort (CoVAC) study [29], an ongoing prospective cohort study examining the longitudinal effects of violence exposure in adolescence on health and social outcomes. The study setting is Luwero District, Uganda, which has a population of nearly 600,000, includes both urban and rural areas, and relies primarily on agriculture [30]. In 2014, our team found that more than 90% of early adolescents experience physical violence from primary school staff in the district [31].

Wave 1 surveys were administered in 2014 to students in Primary 5, 6, and 7 as part of the Good Schools Study [32], a cluster randomized controlled trial (RCT) of a violence prevention program in 42 schools. A two-step selection process was used for the RCT. In Stage One, schools were randomly selected to participate in the trial. In Stage Two, a random sample of Primary 5, 6, and 7 students were invited to participate. Schools were then randomized to the intervention (i.e. the Good School Toolkit) or wait-list control conditions (i.e., usual practice) (details about sampling and recruitment are reported elsewhere [32]). Wave 2 surveys were administered with the same participants who completed Wave 1 surveys four-years later (in 2018). To be eligible in Wave 2, participants needed to have agreed to be re-contacted in Wave 1. At Wave 2, we sought to reach all participants who completed Wave 1 surveys. Of 3,820 students recruited at Wave 1, 90% (n = 3,431) agreed to be re-contacted and 81% (n = 2,773) completed Wave 2 surveys.

Most young people were interviewed in schools, but some were interviewed in other locations (e.g., at home, at work). Interviewers were conducted by researchers who were extensively trained in approaches to interviewing children about violence, including in how to use non-judgmental techniques, respond to distress, pause the interview if needed, and to ensure the interview was conducted in a private place. Interviews were conducted in English or Luganda. All participants were offered counselling and referrals, and referrals were supported by counsellors engaged by the study depending on severity and urgency. Measures to support the researchers and prevent vicarious trauma were in place during data collection.

To generate school variables to assess as effect modifiers, we drew on the dataset of all 3,431 students (both male and female) who completed Wave 1 and Wave 2 surveys (described above), and a dataset of surveys completed by 591 teachers at Wave 1. As with the student surveys, the staff surveys were administered by trained researchers in-person through face-to-face interviews. Answers were programmed into tablet computers (details reported elsewhere [33]).

**Fig 1:**
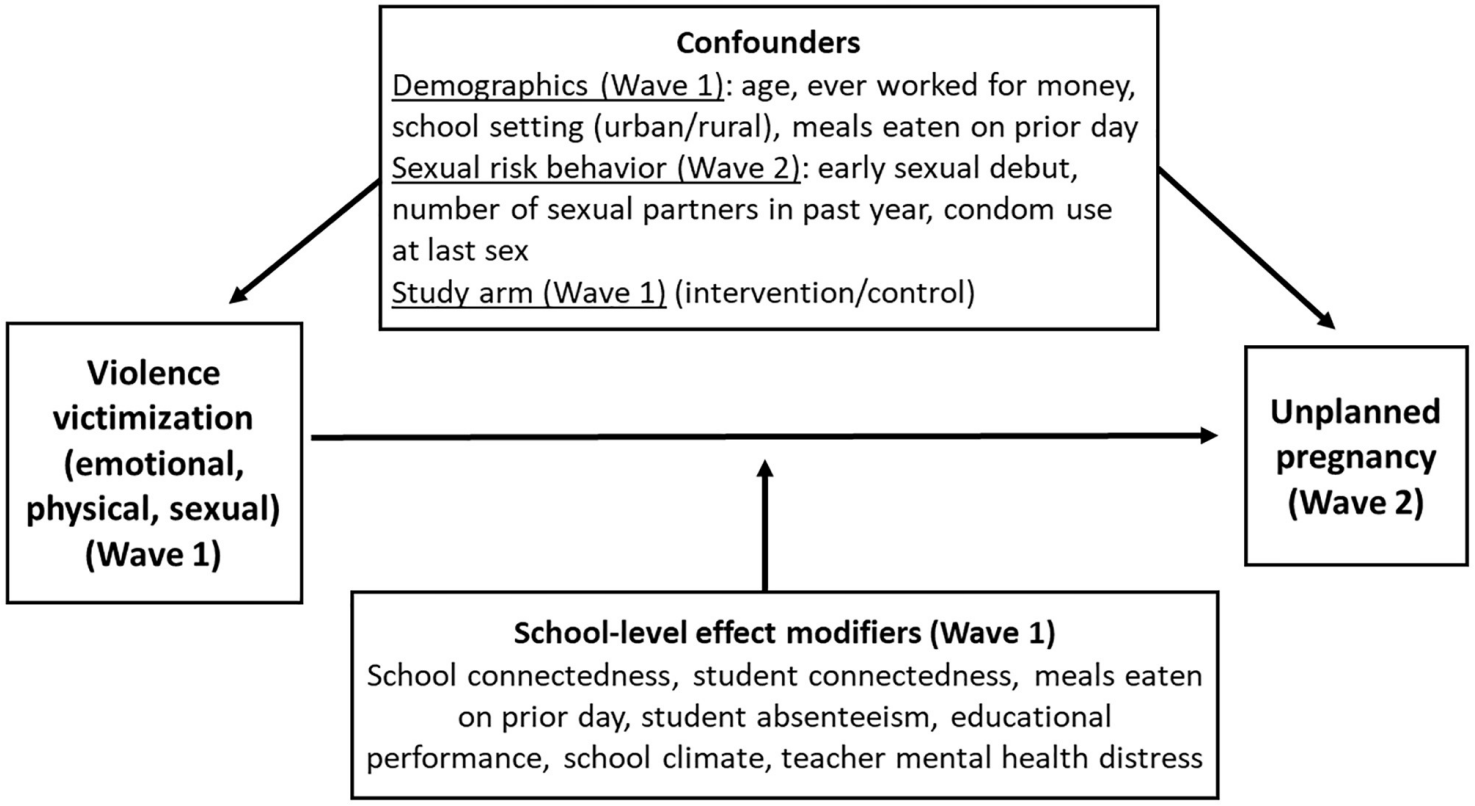
Conceptual framework. Informed analysis of the association between violence victimization and unplanned pregnancy among adolescent girls in Uganda.

We hypothesized that all forms of violence victimization would predict unplanned pregnancy and that these associations would be modified by the school factors examined. Our conceptual framework is in Fig 1. Drawing on existing literature, we adjusted for the following demographic covariates: age [14, 18, 34, 35], employment status (i.e., ever worked for money) [18, 34], rural versus urban setting (in our case, school setting) [18, 35], and socioeconomic status [14, 18, 35, 36], which we assessed via meals eaten on the previous day [37, 38]. In line with previous studies, we also adjusted for sexual risk behavior variables recognized as risk factors for adolescent pregnancy, including early sexual debut [35, 39], number of sexual partners [14, 36, 39], and condom use at last sex [14, 18, 36, 39], in additional to study treatment arm [14]. We selected school-level variables to test as effect modifiers based on their presumed relationship with experiences of violence [21–25] and adolescent pregnancy [26–28].

### Measures

#### Outcome

Unplanned pregnancy, the outcome of interest, was measured at Wave 2. Participants were classified as having experienced an unplanned pregnancy if responding “yes” to, “Have you ever been pregnant?” and “no” to, “Thinking of yours or your partner’s first ever pregnancy, was the pregnancy planned?”

#### Exposure

Violence victimization, the exposure of interest, was measured at Wave 1 using items adapted from the International Society for the Prevention of Child Abuse and Neglect- Child Abuse Screening Tool-Child Institutional (ICAST-CI) [40]. In line with WHO recommendations, violence victimization was assessed using self-reports of multiple specific behavioral acts of violence (e.g., being slapped or hit) [41]. Items assessed lifetime experiences of emotional violence (8 items), physical violence (24 items), and sexual violence (13 items) from teachers, family members (i.e., a parent/caregiver or other adult relative), and peers (S1 Table). Adolescents were classified as having experienced a given form of violence if reporting one or more behavioral act in their lifetime and were compared with adolescents who reported no experience of emotional, physical, or sexual violence of any kind. Items were translated into Luganda, reviewed for their appropriateness by a panel of in-country stakeholders, and cognitively tested and iteratively refined prior to their use [31]. We created the following binary variables:

*Any violence*. We generated a variable for those who had experienced any type of violence (i.e., emotional, physical, and/or sexual) from any perpetrator group (i.e., teachers, peers, and/or family members).

*Type of violence*. We generated three variables to look separately at experiences of emotional, physical, and sexual violence from any perpetrator group.

*Violence from perpetrator groups*. We generated three variables to look at experiences of any type of violence (i.e., emotional, physical, or sexual) from the following groups: teachers, family members, and peers.

*Polyvictimization*. We generated one variable to examine experiences of violence from one, two, or all three perpetrator groups (i.e., teachers, peers, and/or family members).

#### Covariates

Demographic covariates (measured at Wave 1) included age (continuous), ever worked for money (yes/no), setting of the adolescent’s school (rural/urban), and at least 3 meals eaten on the day prior (yes/no) as a marker of socioeconomic status. Sexual behavior covariates included early sexual debut (i.e., before age 15) (no/yes), number of sexual partners in the past 12 months (0 or 1 vs. 2 or more), and condom use during last sex (yes/no). Given the young age of participants at Wave 1 (mean = 12.72, SD = 1.44, years), the sexual behavior covariates were drawn from Wave 2, which were considered more reliable indicators of behavior. We also adjusted for the study group from the Good Schools Study RCT (intervention/control).

#### School-level effect modifiers

Wave 1 data were used for school-level variables. Using data from both male and female students (n = 3,431), we generated two composite measures of connectedness: a 4-item measure of school connectedness (score range: 0–12, Cronbach’s alpha: 0.71) and a 3-item measure of peer connectedness (score range: 0–9, Cronbach’s alpha: 0.51). We aggregated students’ responses to each measure by school, assigned each school a mean score, and dichotomized schools into higher versus lower levels of connectedness. A summary of school-level effect modifiers is in S2 Table.

As a proxy for socio-economic status, we examined the number of meals students reported having eaten the day prior. We generated a mean per school for whether the students reported eating one, two, or three+ meals, and then dichotomized schools into more versus less meals eaten on the day prior by the students surveyed. To examine student absenteeism, we drew on a survey question assessing the number of school days attended in the past week (ranging from 0 to 5+ days). We transformed the variable to examine the number of schooldays missed in the past week, generated a school mean, and dichotomized schools into less versus more absenteeism.

Educational tests—adapted from a trial in Kenya [42]—included word recognition tests in English and Luganda (scored 1–50), timed reading tests in English and Luganda (words per minute), and two reading comprehension activities in English (scored 0–68 and 1–5) and Luganda (scored 0–61 and 1–5). Scores were grouped into quintiles and summed to create a global score. Mean scores were then derived per school and schools were dichotomized into higher versus lower educational performance.

Using Wave 1 data from 591 teachers, we generated variables for school climate and teacher mental health distress. To assess school climate, we summed and aggregated teachers’ responses to 16 items (score range: 0–48, Cronbach’s alpha: 0.78), assigned a mean score to each school, and dichotomized schools into higher/lower perceived quality of school climate [43]. Teacher mental health distress was assessed based on yes/no responses to 20 items from the Self-Report Questionnaire (SRQ)-20 [44] (score range = 0–20, Cronbach’s alpha: 0.71). Each symptom reported was given a score of one. A school mean was generated, and schools were dichotomized into less/more distress.

#### Statistical analysis

We restricted our sample to girls completing Wave 1 and Wave 2 surveys and conducted a complete case analysis. Girls who reported an unplanned pregnancy prior to or during the same year as the Wave 1 survey were excluded from analyses to ensure that the exposure would precede the outcome. We generated descriptive statistics for all variables and bivariate associations with unplanned pregnancy (the outcome). For composite variables other than the violence variables, those responding to less than half of the items were coded as missing. Where more than half of the items were answered, the missing items were replaced by the individual’s mean score on the remaining items.

We used mixed effects logistic regression to obtain crude and adjusted odds ratios for the effects of exposure to violence at Wave 1 on unplanned pregnancy at Wave 2, accounting for the clustering of students within schools. Adjusted analyses included students’ age at Wave 1, study treatment arm, their school’s location (urban/rural), and whether they had worked for money (yes/no), eaten 3 or more meals on the day (yes/no), experienced early sexual debut (<15 years) (yes/no), reported 2 or more sexual partners in the past year (yes/no), and used contraception at last sex (yes/no). Covariates were specified *a priori*. Collinearity was assessed between any pairs of variables using variance inflation factors, with none identified.

Where the association between the school factor and both the violence variable and unplanned pregnancy were significant at the p<0.10 level, we tested for effect modification by the school factor. We added an interaction term between the violence variable and the school factor in the final multivariate model. Analyses were conducted in Stata 15.

#### Ethical considerations

Written informed consent, including written assent from minors, was obtained from all participants. Parents were informed of the study and had the option of withdrawing their children. All adolescents were offered counselling at the end of the interview and a comprehensive child protection plan was implemented to support those needing services. Ethical approval was obtained from the Uganda Virus Research Institute (UVRI and UNCST) (SS2520 and SS4722), the London School of Hygiene and Tropical Medicine Ethics Committee (6183 and 14768), and the University of London (UCL) Institute of Education (IoE) Research Ethics Committee (1091).

## Results

### Descriptive statistics

In total, 1,451 adolescent girls completed surveys at Waves 1 and 2 and had information on all variables of interest. Two adolescent girls reported their first pregnancy in the same year as the year of Wave 1 data collection and were excluded. This resulted in an analysis sample of 1,449 girls, representing 78% of the 1,853 girls who completed Wave 1 surveys. At Wave 1, compared to those who were followed up, those lost to follow-up were older (13.0 versus 12.7 years old, p<0.001) and significantly more likely to have experienced sexual violence (p<0.01) and to be from schools with lower versus higher educational performance (p<0.001) (S3 Table).

At Wave 1, the mean age of the adolescent girls was 12.73 years (inter-quartile range = 12–14). Over half were at urban versus rural schools (55%) and had eaten at least three meals yesterday (58%). Most girls (88%) reported experiencing any emotional, physical, or sexual violence in their lifetimes (Table 1).

**Table 1:** Violence variables, covariates, and effect modifiers, stratified by unplanned pregnancy among adolescent girls (n = 1,449).

	Sample	Unplanned pregnancy (Wave 2)		
	% (n/N)	Yes	No	p value^^
		13.9% (201/1,449)	86.1% (1,248/1,449)	
**Demographics (Wave 1)**				
Age (mean, SD, range)	12.73 (1.44), 8–19	13.65 (1.21), 11–19	12.58 (1.42), 8–17	<0.001
Age group				
8–10 years	7.1% (103/1,449)	0% (0/201)	8.3% (103/1,248)	<0.001
11–14 years	83.6% (1,212/1,449)	79.6% (160/201)	84.3% (1,052/1,248)	
15–19 years	9.3% (134/1,449)	20.5% (41/201)	7.5% (93/1,248)	
Class				
Primary 5				
Primary 6				
Primary 7				
Ever worked for money (yes vs. no)	16.2% (235/1,449)	19.4% (39/201)	15.7% (196/1,248)	0.19
School setting				
Rural	55.1% (798/1,449)	74.6% (150/201)	51.9% (648/1,248)	<0.001
Urban	44.9% (651/1,449)	25.4% (51/201)	48.1% (600/1,248)	
At least 3 meals eaten yesterday (yes vs. no)	57.8% (838/1,449)	67.2% (135/201)	56.3% (703/1,248)	0.004
**Additional demographics (Wave 2)**				
Currently married	2.4% (34/1,449)	6.0% (12/201)	1.8% (22/1,248)	<0.001
What she does most of the time				
Attends primary school	2.3% (33/1,449)	0% (0/201)	2.6% (33/1,248)	<0.001
Attends secondary school	51% (739/1,449)	0.5% (1/201)	59.1% (738/1,248)	
Attends vocational school	5.1% (74/1,449)	3.0% (6/201)	5.5% (68/1,248)	
Does not attend school and working for money	14.3% (207/1,449)	25.4% (51/201)	12.5% (156/1,248)	
Does not attend school and NOT working for money	27.3% (396/1,449)	71.1% (143/201)	20.4% (253/1,248)	
**Sexual risk behavior variables (Wave 2** ^ **)**				
Early sexual debut (<15 years) (yes vs. no)	2.6% (38/1,449)	7.5% (15/201)	1.8% (23/1,248)	<0.001
Number of sexual partners in past year				
0 or 1	31.5% (457/1,449)	74.6% (150/201)	24.6% (307/1,248)	<0.001
2 or more	68.5% (992/1,449)	25.4% (51/201)	75.4% (941/1,248)	
Condom use at last sex				
Yes or N/A	77.4% (1,122/1,449)	22.4% (45/201)	86.3% (1,077/1,248)	<0.001
No	22.6% (327/1,449)	77.6% (156/201)	13.7% (171/1,248)	
**Other covariate (Wave 1)**				
Study arm				
Control	49.0% (711/1,449)	44.3% (89/201)	49.7% 620/1,248)	0.16
Intervention	51.0% (740/1,449)	55.7% (112/201)	50.3% (628/1,248)	
**School-level modifiers (Wave 1)**				
School connectedness				
Higher	50.9% (738/1,449)	61.2% (123/201)	49.3% (615/1,248)	<0.01
Lower	49.1% (711/1,449)	38.8% (78/201)	50.7% (633/1,248)	
Student connectedness				
Higher	53.6% (776/1,449)	67.2% (135/201)	51.4% (641/1,248)	<0.001
Lower	46.5% (673/1,449)	32.8% (66/201)	48.6% (607/1,248)	
Meals eaten on day prior				
More	50.7% (735/1,449)	38.3% (77/201)	52.7% (658/1,248)	<0.001
Less	49.3% (714/1,449)	61.7% (124/201)	47.3% (590/1,248)	
Student absenteeism				
Less	51.7% (749/1,449)	45.8% (92/201)	52.6% (657/1,248)	0.07
More	48.3% (700/1,449)	54.2% (109/201)	47.4% (591/1,248)	
Educational performance				
Higher	52.5% (760/1,449)	32.8% (66/201)	55.6% (694/1,248)	<0.001
Lower	47.6% (689/1,449)	67.2% (135/201)	44.4% (554/1,248)	
School climate				
Higher	53.2% (771/1,449)	58.7% (118/201)	52.3% (653/1,248)	0.09
Lower	46.8% (678/1,449)	41.3% (83/201)	47.7% (595/1,248)	
Teacher mental health distress				
Less	50.0% (724/1,449)	52.7% (106/201)	49.5% (618/1,248)	0.40
More	50.0% (725/1,449)	47.3% (95/201)	50.5% (630/1,248)	
Violence variables* **(Wave 1)**				
Any violence victimization				
No violence of any kind	11.6% (168/1,449)	7.0% (14/201)	12.3% (154/1,248)	0.03
Any emotional, physical, or sexual	88.4% (1,281/1,449)	93.0% (187/201)	87.7% (1,094/1,248)	
*Type of violence*				
Emotional violence	80.9% (711/879)	88.3% (106/120)	79.7% (605/759)	0.03
Physical violence	88.1% (1,246/1,414)	92.9% (182/196)	87.4% (1,064/1,218)	0.03
Sexual violence	26.3% (60/228)	44.0% (11/25)	24.1% (49/203)	0.03
*Perpetrator group*				
Teacher violence	88.0% (1,231/1,339)	92.8% (181/195)	87.2% (1,050/1,204)	0.03
Peer violence	80.1% (677/845)	87.4% (97/111)	79.0% (580/734)	0.04
Family violence	66.8% (338/506)	80.3% (57/71)	64.6% (281/435)	0.01
*Polyvictimization* **				
Violence from:				
No perpetrator groups	11.6% (168/1,449)	7.0% (14/201)	12.3% (154/1,248)	0.13
1 perpetrator group	33.0% (478/1,449)	32.8% (66/201)	33.0% (412/1,248)	
2 perpetrator groups	44.2% (641/1,449)	46.8% (94/201)	43.8% (547/1,248)	
3 perpetrator groups	11.2% (162/1,449)	13.4% (27/201)	10.8% (135/1,248)	

^These variables are from Wave 2 since these were thought to be a better representation of sexual risk behavior given the older age of participants.

^^Chi-square tests for binary/categorical variables and t-tests for continuous variables.

*All violence variables use "no violence of any kind" as the unexposed group (n = 168).

**Perpetrator groups include teachers, peers, and family members.

At Wave 2, 13.9% reported an unplanned pregnancy. Higher proportions of those with an unplanned pregnancy reported nearly every form of violence examined, compared to those without an unplanned pregnancy. Those with an unplanned pregnancy were also significantly more likely to report being married and not attending school or working for money at Wave 2 (Table 1).

Characteristics of school-level effect modifiers are in Table 2. Of note, schools averaged 2.28 on a scale of 1 to 3+ meals eaten yesterday (SD = 0.22) and less than one out of five days for student absenteeism in the past week (mean = 0.34 days, SD = 0.17).

**Table 2:** Associations between lifetime experiences of violence at Wave 1 (2014) and unplanned pregnancy among adolescent girls at Wave 2 (2018)* (n = 1,449).

	n	Crude Odds Ratio	95% CI	p value	Adjusted Odds Ratio**	95% CI	p value
Any violence victimization	1,449	2.05	(1.12–3.77)	0.02	1.99	(1.03–3.85)	0.04
*Type of violence*							
Emotional violence	879	2.22	(1.16–4.27)	0.02	1.95	(0.96, 3.98)	0.07
Physical violence	1,414	2.08	(1.13–3.81)	0.02	1.96	(1.02, 3.79)	0.04
Sexual violence	228	2.82	(1.08–7.36)	0.03	1.72	(0.51–5.80)	0.39
*Perpetrator group*							
Teacher violence	1,399	2.12	(1.15–3.91)	0.02	1.96	(1.01, 3.79)	0.05
Peer violence	845	2.05	(1.08–3.89)	0.03	2.00	(1.00, 4.03)	0.05
Family violence	506	2.30	(1.17–4.52)	0.02	2.23	(1.07–4.65)	0.03
*Polyvictimization* ^							
Violence from:	1,449						
No perpetrator groups		1			1		
1 perpetrator group		1.93	(1.02–3.65)	0.04	2.04	(1.01–4.15)	0.05
2 perpetrator groups		2.09	(1.11–3.93)	0.02	1.90	(0.96–3.77)	0.07
3 perpetrator groups		2.40	(1.16–4.98)	0.02	2.21	(0.99–4.95)	0.05

*All models use "no violence of any kind" as the unexposed group (n = 168).

**Adjusted for: age (continuous), school setting (rural/urban), at least 3 meals eaten yesterday (yes/no), condom use at last sex (no/yes or not applicable), early sexual debut (<15 years) (no/yes), number of sexual partners (0 or 1/2 or more), and study group (intervention/control).

^Perpetrator groups include teachers, peers, and family members.

### Associations between multiple forms of violence victimization (Wave 1) and unplanned pregnancy (Wave 2)

Nearly every type of violence was significantly associated with unplanned pregnancy in crude analyses, many of which remained significant in analyses adjusted for demographics, sexual risk behavior variables, and treatment arm (Table 2).

Girls who had experienced any violence victimization had roughly twice the odds of an unplanned pregnancy (OR = 1.99, 95%CI = 1.03–3.85, p<0.05) compared to girls with no violence experience in adjusted analyses. Similar odds were observed in adjusted analyses for physical violence, violence from each perpetrator group (teachers, peers, and family members), and violence from one and three perpetrator groups compared to no violence exposure. Experiencing emotional violence versus no violence was significantly associated in crude analyses but the p value weakened to 0.07 in adjusted analyses. Experiencing sexual violence versus no violence was also significantly associated in crude analyses but no longer significant in adjusted analyses.

### School factors as modifiers of the association between violence victimization and unplanned pregnancy

Characteristics of school-level effect modifiers are in Table 3. Of school-level factors, crude evidence of an association with violence victimization and unplanned pregnancy was observed for school and peer connectedness, number of meals eaten yesterday, and educational performance; hence, these variables were tested as effect modifiers. Although crude associations for school and peer connectedness were in the opposite direction than expected—whereby the odds of an unplanned pregnancy were higher in schools with higher levels of connectedness—these associations did not hold in adjusted analyses (S4 Table).

**Table 3:** Characteristics of school-level effect modifiers (n = 42 schools).

	Possible range	School mean (standard deviation), range
School connectedness*	0 (low) to 12 (high)	9.76 (0.43), 8.68–10.5
Peer connectedness*	0 (low) to 9 (high)	5.72 (0.45), 4.62–6.73
Meals eaten on day prior*	1 to 3 meals	2.28, (0.22), 1.85–2.75
Days absent in past week	0 to 5 days	0.34 (0.17), 0.04–0.78
Educational performance*	0 (low) to 4 (high)	2.17 (0.46), 1.34–3.12
School climate*	0 (low) to 48 (high)	31.06 (2.55), 26.57–36.69
Teacher mental health problems	0 (low) to 20 (high)	3.89 (0.99), 1.86–5.80

*Variables were recoded in the analysis such that higher scores corresponded with the hypothesized direction for higher odds of unplanned pregnancy.

Evidence of interaction was observed for school and peer connectedness (all p values for interaction were <0.05) (Table 4). In schools with lower levels of both school and peer connectedness, adolescent girls who had experienced violence had greater odds of an unplanned pregnancy compared to those who had not experienced violence. Specifically, those who attended a school with lower school connectedness had over five times the odds of an unplanned pregnancy if they experienced any violence (OR = 5.24, 95% CI = 1.46–18.86), teacher violence (OR = 5.10, 95% CI = 1.41–18.41), or physical violence (OR = 5.10, 95% CI = 1.42–18.34) versus no violence. Additionally, those who attended a school with lower peer connectedness had over 5 times the odds of an unplanned pregnancy if they experienced any violence (OR = 5.53, 95% CI = 1.53–19.98) or teacher violence (OR = 5.44, 95% CI = 1.50–19.73) versus no violence. Those who attended a school with low peer connectedness had between about four and eight times the odds of an unplanned pregnancy if they did versus did not experience polyvictimization (OR for 1 perpetrator group = 8.02, 95% CI = 2.09–30.67; OR for 2 perpetrator groups = 4.44, 95% CI = 1.18–16.74; OR for 3 perpetrator groups = 4.13, 95% CI = 0.84–20.21).

**Table 4:** Adjusted estimates of the odds ratio for the association between violence and unplanned pregnancy, stratified by levels of school and peer connectedness.

	n	High connectedness		Low connectedness		p value for interaction
		Adjusted Odds Ratio^	95%CI	Adjusted Odds Ratio^	95%CI	
School connectedness						
Any violence victimization	1,449	1.24	(0.55, 2.77)	5.24	(1.46, 18.86)	0.05
Teacher violence	1,399	1.24	(0.55, 2.79)	5.10	(1.41, 18.41)	0.05
Physical violence	1,414	1.24	(0.56, 2.77)	5.10	(1.42, 18.34)	0.05
Peer connectedness						
Any violence victimization	1,449	1.17	(0.52, 2.60)	5.53	(1.53, 19.98)	0.03
Teacher violence	1,399	1.15	(0.52, 2.60)	5.44	(1.50, 19.73)	0.03
Polyvictimization*	1,449					
No perpetrator groups		1		1.00		0.03
1 perpetrator group		0.98	(0.41, 2.34)	8.02	(2.09, 30.67)	
2 perpetrator groups		1.20	(0.52, 2.78)	4.44	(1.18, 16.74)	
3 perpetrator groups		1.54	(0.58, 4.06)	4.13	(0.84, 20.21)	

^Adjusted for age (continuous), school setting (rural/urban), at least 3 meals eaten yesterday (yes/no), condom use at last sex (no/yes or not applicable), early sexual debut (<15 years) (no/yes), number of sexual partners (0 or 1/2 or more), and study group (intervention/control). p values are Likelihood Ratio Tests.

*Perpetrator groups include teachers, peers, and family members.

In contrast, for girls in schools with higher levels of school and peer connectedness, those who experienced violence were not any more likely to have an unplanned pregnancy compared to those who did not experience these forms of violence. Of note, the confidence intervals were wide for the low connectedness odds ratios.

## Discussion

Our findings strongly support our hypothesis that early adolescent violence victimization predicts subsequent unplanned pregnancy among adolescent girls in Luwero District, Uganda. Specifically, we found that any experience of emotional, physical, or sexual violence—in addition to violence from teachers, peers, and family members, physical violence, and violence from one or three perpetrator groups—resulted in a roughly two-fold increase in the odds of unplanned pregnancy compared to no violence victimization. These results strengthen existing literature on the association [12–15, 17] by providing longitudinal evidence from a LMIC context which was previously absent and by helping to fill important gaps in our understanding of the forms of violence victimization which predict pregnancy in adolescents. Importantly, we observed a high prevalence of violence victimization (88% for any violence) and unplanned pregnancy (14%) at Wave 1 among adolescent girls in this Ugandan setting. The cultural normativeness hypothesis supposes that where forms of violence are normative or accepted, they are less likely to be associated with adverse outcomes [45]. However, our study shows that violence remains an important predictor of an adverse outcome—i.e., unplanned pregnancy—despite its high prevalence in the population and the cultural normativeness hypothesis.

Our findings for the association between unplanned pregnancy and violence from each of the three perpetrator groups examined (i.e., teachers, peers, and family members) is notable. Of existing studies, none to our knowledge has examined the perpetrators of violence as they relate to adolescent pregnancy. These findings highlight the importance of reducing violence in schools, in homes, and among peer groups to help prevent unplanned pregnancy and support calls in the literature for more evidence-based violence prevention interventions in these settings, particularly in LMIC [46, 47]. Interventions like the school-based Good School Toolkit in Uganda [32] and the home-centered Parenting for Lifelong Health (Sinovuyo Teen) program in South Africa [48] may play a key role in these efforts.

Our findings add to the literature on the patterns of risk resulting from distinct types of violence victimization. We found a stronger association between physical violence and unplanned pregnancy (roughly two-times the odds) compared to an odds ratio of about 1.5 in the 2014 meta-analysis of abuse history and adolescent pregnancy [12]. Whereas multiple studies have found associations between sexual violence and adolescent pregnancy [12, 13, 16, 17], we only observed sexual violence to be associated with unplanned pregnancy in crude analyses. The absence of significant associations in adjusted analyses may be due to the lower prevalence of sexual violence at Wave 1 (when most participants were aged 11–14 years) compared to other forms of violence in our study. Associations between emotional violence and adolescent pregnancy have also been identified in some studies [13], but we found only weak evidence of an association in adjusted analyses (p = 0.07). Furthermore, we only observed associations for some forms of polyvictimization (i.e., violence from one and three but not two perpetrator groups), although all associations were in the hypothesized direction. Future studies should examine the severity of violence as it relates to unplanned pregnancy, which has been the subject of some previous studies [12] but was outside of the scope of the current analysis.

Of note, we identified school and peer connectedness as effect modifiers of the association between violence victimization and adolescent pregnancy. While the confidence intervals in our interaction analysis were wide, a stronger association between experience of violence and unplanned pregnancy was observed for girls in schools with lower versus higher levels of school and peer connectedness. Both school connectedness [49, 50] and peer connectedness [51] have been linked to improved outcomes for students in such areas as academics, attendance, socialization, and emotional and mental wellbeing. It may be that the positive environmental attributes of schools with higher levels of school and peer connectedness can mitigate the negative implications of violence exposure, thus disrupting the link between violence victimization and adolescent pregnancy. These findings place a spotlight on the importance of enhancing feelings of safety, belonging, care, and enjoyment at school, in addition to closeness and support from peers. Interventions which take a whole-school approach towards enhancing the school environment and reducing violence—such as the Good School Toolkit in Uganda [32], Right to Play’s intervention in Pakistan [52], and The Irie Classroom Toolbox in Jamaica [53]—may achieve these goals. However, existing interventions could be strengthened by integrating a greater focus on school and peer connectedness. Given the wide confidence intervals in our interaction analysis, future studies should seek to replicate these findings.

Interestingly, we did not identify school climate—a related construct [21]—as an effect modifier. This may have been due to deficiencies with the measure we developed, even though we piloted the survey items and the composite measure showed good internal consistency. School climate is also a broader construct and was measured from the perspective of teachers, while connectedness is more focused on emotional aspects and was measured from the perspective of students, which could indicate that feelings of safety and belonging among students are more important than other aspects of the school climate.

Strengths of this study include its use of longitudinal data from a large sample in an under-researched location, its high response rates, and its low levels of missing data. Study limitations must also be noted. We observed differences at Wave 1 in age and experience of sexual violence among those lost compared to those followed up, but a complete case analysis was still deemed valid since the association between violence and adolescent pregnancy was not thought to differ between the groups. If the association did differ for those lost to follow-up, we expect it may have resulted in a stronger association between violence and unplanned pregnancy, which would make our results conservative. Although data collectors received thorough training and our violence measures incorporated multiple behaviorally specific acts, social desirability bias and/or recall bias may have occurred given that our measures of violence and unplanned pregnancy were by necessity self-reported. Given the stigma associated with violence, we would expect some under-reporting [54]. Our interaction analysis of school factors should be replicated with a larger sample given the wide confidence intervals and given the possibility that school factors may have changed over the four years of the study, though we expect school factors to be constant. Finally, we used Wave 1 data on violence victimization as a predictor for unplanned pregnancy four years later. It may be that experiences of violence after Wave 1 modify the association between violence at Wave 1 and unplanned pregnancy at Wave 2, which was outside of the scope of this study but could be an avenue for future research.

## Conclusions

This longitudinal study strongly supports evidence for an association between violence victimization and unplanned pregnancy among adolescent girls. It offers valuable data on the forms of violence victimization which predict unplanned pregnancy in a sub-Saharan African setting, with a particular contribution of new knowledge about perpetrator types (i.e., teachers, peers, and family members). These findings have implications for policymakers, underscoring the importance of recognizing the widespread experiences and consequences of violence among adolescent girls which are often overlooked in policy and practice [54]. Interventions addressing violence in multiple contexts, with a focus on the school and home, may be critical to preventing unplanned pregnancy in adolescence and thus minimizing other detrimental health and social outcomes for girls as they reach adulthood. Of note, our study identifies two school factors—school and peer connectedness—which modify the association between violence victimization and unplanned pregnancy, offering a target point for interventions in the school environment to interrupt this pathway. Future research should further examine perpetrator types as they relate to adolescent pregnancy to enhance our understanding of where and by whom the violence victimization occurs. They should also assess associations with the type of violence (i.e., physical, emotional, sexual) since our lack of significant adjusted associations in some cases was unexpected and consider the severity of violence as linked to adolescent pregnancy. Finally, future studies should further examine school-level variables as effect modifiers of the association given the wide confidence intervals in our study and additional experiences of violence as potential effect modifiers of the association between violence in early adolescence and unplanned pregnancy in late adolescence.

## Supporting information

S1 Table Description of violence measures. Details about measure definitions and coding.
(DOCX)

S2 Table Description of school-level effect modifiers. Details about measure definitions and coding.
(DOCX)

S3 Table Characteristics of study participants followed up versus lost to follow-up. (DOCX)

S4 Table Associations between school-level variables at Wave 1 (2014) and unplanned pregnancy among adolescent girls at Wave 2 (2018)* (n = 1,449). (DOCX)
