# Supplementary material for: The association between violence victimization and subsequent unplanned pregnancy among adolescent girls in Uganda: Do primary schools make a difference?
Source: PLOS Glob Public Health. 2023 Jul 31;3(7):e0001141. doi: 10.1371/journal.pgph.0001141 (PMC10389730; doi:10.1371/journal.pgph.0001141)
Supplement: S1 Table — Details about measure definitions and coding. (DOCX) [file pgph.0001141.s001.docx]

**The association between violence victimization and unplanned pregnancy among adolescent girls: Do primary school factors make a difference?**

**S1 Table. Description of violence measures.** Details about measure definitions and coding.

| Any violence | One variable generated by aggregating reports of emotional, physical, and sexual violence from any perpetrator group (i.e. teachers, peers, or family members; see items below). | Coded 1 if “Yes” to any reports of emotional, physical, or sexual violence; coded 0 if no reports of violence of any kind |
| --- | --- | --- |
| Teacher violence* | One variable generated based on the following survey items:  Has a teacher or other adult that works at your school ever done any of the following things to you?  Emotional violence: (1) Cursed, insulted, shouted at or humiliated you? (2) Referred to your skin colour/gender/religion/tribe or health problems you  have in a hurtful way? (3) Stopped you from being with other children to  make you feel bad or lonely? (4) Tried to embarrass you because you were an orphan or without a parent? (5) Embarrassed you because you were unable to buy things? (6) Stole or broke or ruined your belongings? (7) Threatened you with bad marks that you didn’t deserve? (8) Accused you of witchcraft?  Physical violence: (1) Hurt you or caused pain to you? (2) Slapped you with a hand on your face or head as punishment? (3) Slapped you with a hand on your arm or hand? (4) Twisted your ear as punishment? (5) Twisted your arm as punishment? (6) Pulled your hair as punishment? (7) Hit you by throwing an object at you? (8) Hit you with a closed fist? (9) Hit you with a stick? (10) Caned you? (11) Kicked you? (12) Knocked you on the head as punishment? (13) Made you dig, slash a field, or do other labor as punishment? (14) Hit your fingers or hands with an object as punishment? (15) Crushed your fingers or hands as punishment? (16) Made you stand/kneel in a way that hurts to punish you? (17) Made you stay outside for example in the heat or rain to punish you? (18) Burnt you as punishment? (19) Taken your food away from you as punishment? (20) Forced you to do something that was dangerous? (21) Choked you? (22) Tied you up with a rope or belt at school? (23) Tried to cut you purposefully with a sharp object? (24) Severely beat you up?  Sexual violence: (1) Teased you or made sexual comments about your breasts, genitals, buttocks or other body parts? (2) Touched your body in a sexual way or in a way that made you uncomfortable? (3) Showed you pictures, magazines, or movies of people or children doing sexual things? (4) Made you take your clothes off when it was not for a medical reason? (5) Opened or took their own clothes off in front of you when they should not have done so? (6) Kissed you when you didn’t want to be kissed? (7) Made you touch their genitals, breasts or buttocks when you didn’t want to? (8) Touched your genitals, breasts or buttocks when you didn’t want them to? (9) Gave you money/things to do sexual things? (10) Involved you in making sexual pictures or videos? (11) Threatened or pressured you to have sex or do sexual things with them? (12) Actually made you have sex with them by threatening or pressuring you, or by making you afraid of what they might do? (13) Made you have sex with them by physically forcing you? | Coded 1 if “Yes” to any of the items (from teachers/school staff); coded 0 if no reports of violence of any kind |
| Peer violence | One variable generated based on the following survey items:  Has anyone besides a school staff member ever:  Emotional violence/neglect: (1) Insulted you, or called you rude or hurtful names? (2) Accused you of witchcraft? (3) Locked you out or made you stay outside? (4) Not given you food?  Physical violence: (1) Twisted your arm or any other body part, slapped you, pushed you or thrown something at you? (2) Punched you, kicked you, or hit you with a closed fist? (3) Hit you with an object, such as a stick or a cane, or whipped you? (4) Cut you with a sharp object or burnt you?  Sexual violence: (1) Disturbed or bothered you by making sexual comments about you? (2) Kissed you, when you did not want them to? (3) Touched your genitals or breasts when you did not want them to, or in a way that made you uncomfortable? (4) Threaten or pressure you to make you do something sexual with them? (5) Make you have sex with them, because they threatened or pressured you? (6) Had sex with you, by physically forcing you? | Coded 1 if “Yes” to any of the items and “male student” or “female student” selected when asked “Who did this to you?”; coded 0 if no reports of violence of any kind |
| Family violence | One variable generated, using the same items as for peer violence above. | Coded 1 if “Yes” to any of the items and “parent/caregiver” or “other adult relative” selected when asked “Who did this to you?”; coded 0 if no reports of violence of any kind |
| Types of violence | Three variables generated by aggregating reports of the following types of violence from any perpetrator groups (i.e. teachers, peers, or family members):   1. Emotional violence 2. Physical violence 3. Sexual violence | For each variable, coded 1 if “Yes” to the forms of violence indicated; coded 0 if no reports of violence of any kind |
| Poly-victimization | One variable generated as a categorical variable with four levels, aggregating data from across the three perpetrator groups (i.e. teachers, peers, and family members):   1. No violence from any perpetrator group 2. Violence from one perpetrator group 3. Violence from two perpetrator groups 4. Violence from all three perpetrator groups | Coded as 0 for “no violence from any perpetrator group,” 1 for “violence from 1 group,” 2 for “violence from 2 groups,” and 3 for “violence from all 3 groups” |

*More questions were asked about teacher violence than violence from other groups given that teacher/staff violence was the primary outcome in the Good Schools Study.
