# Supplementary material for: The association between violence victimization and subsequent unplanned pregnancy among adolescent girls in Uganda: Do primary schools make a difference?
Source: PLOS Glob Public Health. 2023 Jul 31;3(7):e0001141. doi: 10.1371/journal.pgph.0001141 (PMC10389730; doi:10.1371/journal.pgph.0001141)
Supplement: S2 Table — Details about measure definitions and coding. (DOCX) [file pgph.0001141.s002.docx]

**The association between violence victimization and unplanned pregnancy among adolescent girls: Do primary school factors make a difference?**

**S2 Table. Description of school-level effect modifiers.** Details about measure definitions and coding.

| School connectedness | One variable generated from 4 items. Response options included: ‘all the time,’ ‘most of the time,’ ‘sometimes,’ ‘never’:   1. I feel that my teachers care about me. 2. I feel safe in school. 3. I feel like I belong at school. 4. I like to spend time at school. | Scores summed, modelled as a continuous variable. Range 0 (low) to 12 (high). Reverse coded so that higher scores would correspond to lower levels of school connectedness.  School means calculated and schools dichotomised into higher or lower connectedness at 50%.  In our study, the Cronbach’s alpha was 0.71. |
| --- | --- | --- |
| Peer connectedness | One variable generated from 3 items. Response options included: ‘all the time,’ ‘most of the time,’ ‘sometimes,’ ‘never’:   1. I feel close to students at my school. 2. I have friends that I can talk to about important things. 3. I have friends that I can count on for support. | Scores summed, modelled as a continuous variable. Range 0 (low) to 9 (high). Reverse coded so that higher scores would correspond to lower levels of peer connectedness.  School means calculated and schools dichotomised into higher or lower connectedness at 50%. In our study, the Cronbach’s alpha was 0.51. |
| Meals eaten on day prior | One variable generated using a single survey item:  Yesterday, how many meals did you eat?   1. One meal 2. Two meals 3. Three or more meals | Coded as 1 for “1 meal,” 2 for “2 meals,” and 3 for “3 or more meals.” Reverse coded so that higher scores would correspond with lower numbers of meals eaten. School means calculated and schools dichotomised into higher or lower number of meals eaten at 50%. |
| Student absenteeism | One variable generated using a single survey item:  In the past week, on how many days did you attend school?   1. 0 days 2. 1 day 3. 2 days 4. 3 days 5. 4 days 6. 5 or more days | Coded as 0 for “0 days,” 1 for “1 day,” 2 for “2 days,” 3 for “3 days,” 4 for “4 days,” and 5 for “5 or more days.” Reverse coded to examine the number of days absent instead of present. School means calculated and schools dichotomised into higher or lower absenteeism at 50%. |
| Educational Performance | One variable generated based on the following:   - Word recognition tests in English and Luganda (scored 1-50) - Timed reading tests in English and Luganda (words per minute) - Two reading comprehension activities in English (scored 0-68 and 1-5) and Luganda (scored 0-61 and 1-5). | Scores grouped into quintiles and summed to create a global score. Mean scores were then derived per school and schools were dichotomized into higher/lower educational performance at 50%. |
| School climate | One variable generated from 16 items. Response options included: ‘all the time,’ ‘most of the time,’ ‘sometimes,’ ‘never’:   1. In your opinion, do you have enough opportunities to say what you think and contribute to how the school is run? 2. Do students in your school have an opportunity to say what they think? 3. Do students in your school have an opportunity to contribute to how the school is run? 4. Do you feel that your views on how the school's policies could be improved are welcomed? 5. How often do you take any actions to change how your school is run? 6. Do you feel that there is anybody at your school you can talk to if you feel unhappy about work? 7. Thinking about your school as a whole, do you feel like you are part of a team? 8. Do you have regular staff meetings? 9. Would you say that students feel comfortable talking to you/want to confide in you if they are unhappy about something at home or at school? 10. Do you feel that students respect their peers and adults? 11. Do you feel that school staff respect their students? 12. Do you have a good relationship with the students? 13. Do you have a good relationship with parents? 14. Do you feel concerned about how other school staff members behave at school? (reverse coded) 15. How often does this school experience problems with physical violence? (reverse coded) 16. How often does this school experience problems with bullying (e.g. verbal abuse of staff or students)? (reverse coded) | Range of possible scores was 0 to 48, with higher scores indicating a *less* favorable perception. Items selected with reference to existing school climate scales: School Climate Survey ^1^, Charles F. Kettering Ltd. School Climate Profile ^2^, California School Climate and Survey-Short Form ^3^, and the SRS Safe Schools Survey ^4^. A school mean was generated, and schools were dichotomized into higher/lower school climate at 50%. In our study, the Cronbach’s alpha was 0.78. |
| Teacher mental health distress | One variable generated from 20 items from the Self-Report Questionnaire-20. Response options included Yes=1 and No=0:   1. Do you often have headaches? 2. Is your appetite poor? 3. Do you sleep badly? 4. Are you easily frightened? 5. Do your hands shake? 6. Do you feel nervous, tense, or worried? 7. Is your digestion poor? 8. Do you have trouble thinking clearly? 9. Do you feel unhappy? 10. Do you cry more than usual? 11. Do you find it difficult to enjoy your daily activities? 12. Do you find it difficult to make decisions? 13. Is your daily work suffering? 14. Are you able to play a useful part in life? 15. Have you lost interest in things? 16. Do you feel that you are a worthless person? 17. Has the thought of ending your life been on your mind? 18. Do you feel tired all the time? 19. Do you have uncomfortable feelings in your stomach? 20. Are you easily tired? | Each symptom reported was given a score of one. A school mean was generated (range of 0=low to 20=high distress), and schools were dichotomized into less/more distress at 50%. In our study, the Cronbach’s alpha was 0.71. |
