# Supplementary material for: The association between violence victimization and subsequent unplanned pregnancy among adolescent girls in Uganda: Do primary schools make a difference?
Source: PLOS Glob Public Health. 2023 Jul 31;3(7):e0001141. doi: 10.1371/journal.pgph.0001141 (PMC10389730; doi:10.1371/journal.pgph.0001141)
Supplement: S3 Table — (DOCX) [file pgph.0001141.s003.docx]

| **The association between violence victimization and unplanned pregnancy among adolescent girls: Do primary school factors make a difference?**  **S3 Table. Characteristics of study participants followed up versus lost to follow-up^** | | | |
| --- | --- | --- | --- |
|  | **Followed up 1,449 (78.1%)** | **Lost to follow-up 402 (21.7%)** | **p value** |
| **Demographics** |  |  |  |
| Age (mean, SD, range) | 12.7 (1.44), 8-19 | 13.0 (1.38), 10-17 | <0.001 |
| Ever worked for money (yes vs. no) | 235 (16.2%) | 70 (17.4%) | 0.58 |
| School setting |  |  |  |
| Rural | 798 (55.1%) | 231 (57.5%) | 0.39 |
| Urban | 651 (44.9%) | 171 (42.5%) |  |
| At least 3 meals eaten yesterday (yes vs. no) | 609 (42.1%) | 156 (38.8%) | 0.24 |
| **Violence variables** |  |  |  |
| Any violence victimization |  |  |  |
| No violence of any kind | 168 (11.6%) | 50 (12.4%) | 0.64 |
| Any emotional, physical, or sexual | 1,281 (88.4%) | 352 (87.6%) |  |
| *Type of violence* |  |  |  |
| Emotional violence | 711 (80.9%) | 202 (80.2%) | 0.80 |
| Physical violence | 1,246 (88.1%) | 343 (87.3%) | 0.65 |
| Sexual violence | 60 (26.3%) | 36 (41.9%) | <0.01 |
| *Perpetrator group* |  |  |  |
| Teacher violence | 1,231 (88.0%) | 338 (87.1%) | 0.64 |
| Peer violence | 677 (80.1%) | 182 (78.5%) | 0.58 |
| Family violence | 338 (66.8%) | 96 (65.8%) | 0.81 |
| *Polyvictimization*** |  |  |  |
| Violence from: |  |  |  |
| No perpetrator groups | 168 (11.6%) | 50 (12.4%) | 0.65 |
| 1 perpetrator group | 478 (33.0%) | 138 (34.3%) |  |
| 2 perpetrator groups | 641 (44.2%) | 164 (40.8%) |  |
| 3 perpetrator groups | 162 (11.2%) | 50 (12.4%) |  |
| **Other** |  |  |  |
| Study arm |  |  |  |
| Control | 709 (48.9%) | 198 (49.3%) | 0.91 |
| Intervention | 740 (51.0%) | 204 (50.8%) |  |

^Primary reasons for loss to follow-up included: participants being unable to be contacted, no longer interested, or deceased; participants moving to a distant district or out of the country; participants having cognitive difficulties; and caregivers opting out their children.
