# Supplementary material for: The association between violence victimization and subsequent unplanned pregnancy among adolescent girls in Uganda: Do primary schools make a difference?
Source: PLOS Glob Public Health. 2023 Jul 31;3(7):e0001141. doi: 10.1371/journal.pgph.0001141 (PMC10389730; doi:10.1371/journal.pgph.0001141)
Supplement: S4 Table — (DOCX) [file pgph.0001141.s004.docx]

**The association between violence victimization and unplanned pregnancy among adolescent girls: Do primary school factors make a difference?**

| **S4 Table. Associations between school-level variables at Wave 1 (2014) and unplanned pregnancy among adolescent girls at Wave 2 (2018)* (n=1,449)** | | | | | | |  |
| --- | --- | --- | --- | --- | --- | --- | --- |
|  | **Crude Odds Ratio** | **95%CI** | **p value** | **Adjusted Odds Ratio^** | **95%CI** | **p value** |  |
|  |  |  |  |  |  |  |  |
| School connectedness^ |  |  |  |  |  |  |  |
| Higher | 1 |  |  | 1 |  |  |  |
| Lower | 0.59 | (0.38, 0.92) | 0.02 | 0.78 | (0.57, 1.07) | 0.12 |  |
| Peer connectedness^^ |  |  |  |  |  |  |  |
| Higher | 1 |  |  | 1 |  |  |  |
| Lower | 0.51 | (0.33, 0.78) | 0.002 | 0.75 | (0.51, 1.10) | 0.14 |  |
| Meals eaten on day prior^^^ |  |  |  |  |  |  |  |
| More | 1 |  |  | 1 |  |  |  |
| Less | 1.86 | (1.20, 2.87) | 0.01 | 1.53 | (1.11, 2.12) | 0.01 |  |
| Student absenteeism^^ |  |  |  |  |  |  |  |
| Less | 1 |  |  | 1 |  |  |  |
| More | 1.28 | (0.81, 2.03) | 0.29 | 1.04 | (0.73, 1.49) | 0.82 |  |
| Educational performance^^ |  |  |  |  |  |  |  |
| Higher | 1 |  |  | 1 |  |  |  |
| Lower | 2.36 | (1.70, 3.28) | <0.001 | 2.20 | (1.51, 3.20) | <0.001 |  |
| School climate^ |  |  |  |  |  |  |  |
| Higher | 1 |  |  | 1 |  |  |  |
| Lower | 0.78 | (0.49, 1.23) | 0.28 | 0.85 | (0.61, 1.18) | 0.34 |  |
| Teacher mental health distress^ |  |  |  |  |  |  |  |
| Lower | 1 |  |  | 1 |  |  |  |
| Higher | 0.90 | 0.56, 1.43) | 0.65 | 0.90 | (0.66, 1.23) | 0.52 |  |
| ^Adjusted for school setting (rural/urban) and school mean for number of meals eaten yesterday (higher/lower)  ^^Adjusted for school setting (rural/urban), school mean for number of meals eaten yesterday (higher/lower), early sexual debut (<15 years) (no/yes), number of sexual partners in past year (0 or 1 versus 2 or more), and condom use at last sex (yes or N/A versus no)  ^^^Adjusted for school setting (rural/urban) only | | | | | | |  |
